# Photic Niche Explains Avian Behavioral Responses to Solar Eclipses

**DOI:** 10.1002/ece3.73090

**Published:** 2026-02-10

**Authors:** Neil A. Gilbert, Brent S. Pease, MaryKay Severino, Henry 'Trae' Winter

**Affiliations:** ^1^ Department of Biology Oklahoma State University Stillwater Oklahoma USA; ^2^ School of Forestry and Horticulture Southern Illinois University Carbondale Illinois USA; ^3^ ARISA Lab LLC Medford Massachusetts USA

**Keywords:** acoustics, behavioral ecology, diel activity patterns, participatory science, sensory ecology

## Abstract

Solar eclipses rapidly alter abiotic conditions and thus represent natural experiments for understanding how animals respond to ephemeral environmental change. Using a large acoustic dataset (181 species, 873 locations) from participatory science, we quantified how birds changed their vocalizations in response to the 2023 annular and 2024 total eclipses in North America. During the total eclipse, most species vocalized less, but nocturnal and large‐eyed species vocalized more. The generalized quieting was restricted to locations with > 94% solar obscuration; most bird species vocalized more in locations with 70%–93% solar obscuration, which experienced only modest dimming. During the annular eclipse (which occurred during the nonbreeding season and reached a maximum of 90% obscuration), most birds did not change their vocalization behavior. Thus, changing photic conditions during solar eclipses are reflected in the composition of species vocalizing, leading to ephemeral shifts in ecological soundscapes.

## Graphical abstract

Most bird species—except for those that are adapted for activity under dim conditions—were less likely to vocalize during the maximum of a total solar eclipse, whereas an annular eclipse reaching only 90% solar obscuration did not lead to behavioral effects.

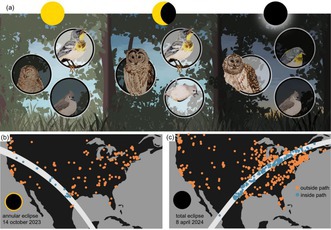

## 1 Introduction

Light is arguably *the* most important environmental variable that structures the lives of organisms (Hut et al. 2012). Light–dark cycles entrain daily behaviors such as activity, foraging, and rest (Prugh and Golden 2014; Russ et al. 2015) as well as seasonal behaviors and life‐history events such as migration and reproduction (Berthold 1996; Hazlerigg and Wagner 2006). Thus, light represents a critical niche dimension, and yet ecological research largely neglects the photic niche in favor of its proxy, time (Kronfeld‐Schor and Dayan 2003; Post 2019; Yerushalmi and Green 2009). Solar eclipses offer rare natural experiments to decouple light levels from their typical time schedules, providing insight into the photic niches of organisms (Aguilar et al. 2025; Gai et al. 2023).

Solar eclipses have held cultural significance throughout human history (Nothaft 2024; Littmann and Espenak 2023), and natural scientists have studied animal responses to eclipses for literally centuries (Wheeler et al. 1935). However, until recently, the effects of eclipses on free‐living animals were the subject of anecdote rather than quantification. For example, Wheeler et al. (1935) provided a narrative of how crepuscular and nocturnal species became active while diurnal species ceased activity during the 1932 eclipse in the northeastern United States (Wheeler et al. 1935). The effort represents a notable early example of participatory science: before the eclipse, the authors solicited observations from the public in newspaper articles and radio broadcasts, resulting in nearly 500 behavioral descriptions of animals ranging from insects to mammals.

Across animal taxa, solar eclipses motivate behaviors resembling those associated with the transition from daytime to dusk or night (Wheeler et al. 1935). For example, observational and quantitative studies alike have documented reductions in activity among diurnal mammals, birds, reptiles, and insects, alongside increased activity in nocturnal or crepuscular taxa during eclipse totality (Brinley Buckley et al. 2018; Galen et al. 2019; Hartstone‐Rose et al. 2020; Wheeler et al. 1935). Typical responses of non‐bird taxa include resting or sheltering initiation for diurnal species and increased activity by nocturnal species during totality. For example, Sinu et al. (2024) documented reduced activity of bees during the period of totality compared with non‐eclipse days. Similarly, orb‐weaving spiders have been documented rapidly deconstructing and reconstructing webs as an eclipse occurred (Uetz et al. 1994). Together, the literature suggests marked responses to rapid changes in light levels, reinforcing the idea that eclipses can be used as natural experiments to test hypotheses about the photic niche.

Recent advances in automated monitoring, expanded participatory‐science networks, and machine‐learning species identification models have enabled quantitative assessments of eclipse responses in animals, especially birds (Brinley Buckley et al. 2018; Kahl et al. 2021; Mann et al. 2025; Nilsson et al. 2018; Oestreich et al. 2024). For example, using radar imagery from a 2017 total eclipse, Nilsson et al. (2018) demonstrated that airborne biological activity (i.e., birds, bats, and insects) declined as totality approached, with some locations demonstrating bursts of activity during totality, consistent with shifts toward nocturnal‐like behavior. Similarly, Hartstone‐Rose et al. (2020) documented contrasting responses of diurnal and nocturnal birds during the 2017 eclipse, with behavioral changes consistent with rapid transitions to dusk‐ or night‐like conditions. Using data from the participatory science program Haikubox, Mann et al. (2025) showed that bird vocalization activity decreased during the 2024 eclipse but did not quantify among‐species variability in eclipse responses (Mann et al. 2025).

Most recently, Aguilar et al. (2025) analyzed community‐science observations of bird behavior (not identified to species) and an acoustic dataset (14 locations) during the 2024 total solar eclipse. They documented higher rates of vocalization immediately before and following totality relative to a normal day but lower rates during totality itself. Importantly, they also showed that species with robust dawn choruses were more likely to alter vocal behavior during eclipses, highlighting the role of circadian and photic sensitivity in shaping responses (Aguilar et al. 2025). This work represents a major contribution in understanding eclipse‐driven vocal dynamics and provides notable evidence that bird responses to eclipses are structured by underlying photic niches.

**FIGURE 1:**
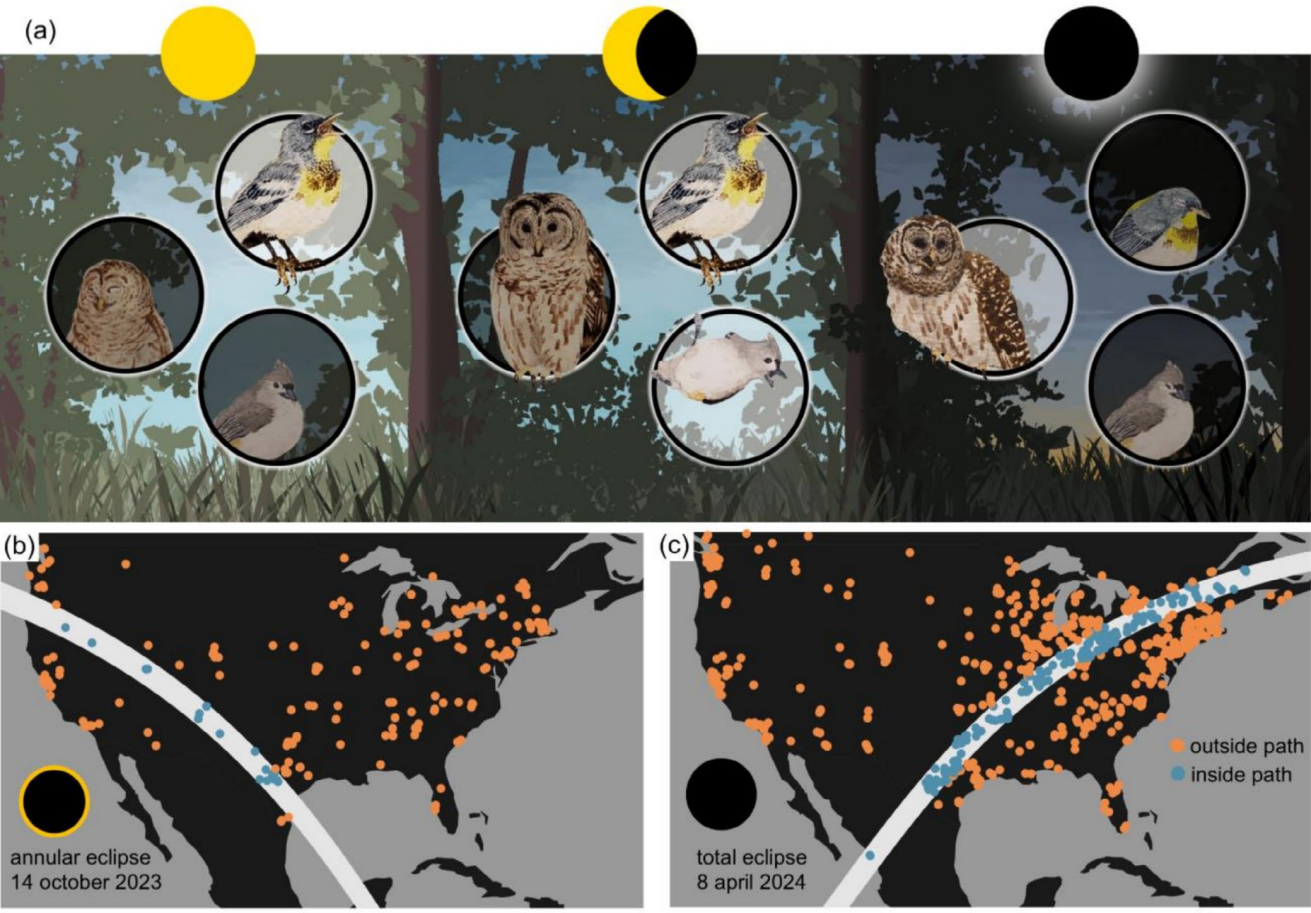
Automated, participatory data reveal avian vocal responses to solar eclipses. (a) We hypothesized that changing light levels drive changes in the vocal profile of avian assemblages. We expected that diurnal species, such as Northern Parula and Tufted Titmouse, would show increased vocalizations leading up to the eclipse before falling silent. In contrast, we expected that nocturnal species like the Barred Owl would show increased vocalizations only during the eclipse maximum. (b–c) Locations of acoustic sensors relative to the paths for the 14 October 2023 annular eclipse (b) and the April 8, 2024, total eclipse (c).

Building on this growing body of research, our objective is to synthesize avian behavioral responses across species, contexts, and eclipses to understand why some species respond more strongly than others. Specifically, we provide a quantitative, trait‐based synthesis of bird vocalization responses to two recent solar eclipses, integrating ecology, morphology, species traits, and environmental context (Figure 1a). By linking eclipse responses to species' traits associated with light sensitivity and exposure, we aim to generalize eclipse effects beyond individual case studies.

Our overarching hypothesis was that species traits and environmental context relating to species' photic niche and light exposure underpin among‐species variability in eclipse responses. Regarding traits, we predicted (P1) that species associated with dim light levels—i.e., nocturnal species and diurnal species that vocalize early in the morning—would show increases in vocalization during eclipses but that species associated with high light levels—i.e., diurnal species, particularly those that vocalize throughout the day—would show decreases (Aguilar et al. 2025; Mann et al. 2025; Wheeler et al. 1935). Similarly, because large‐eyed species are active under dimmer conditions than small‐eyed species (Ausprey 2021; Ockendon et al. 2009; Thomas et al. 2002), we predicted (P2) that species with large eyes would be more likely to vocalize during eclipses. Because nest structure can shield birds from photic cues (Raap et al. 2018), we predicted (P3) that cavity‐nesting species would show a delay in resuming activity post‐totality compared with species with open nests, under the assumption that birds would enter cavities to roost and fail to sense the restoration of sunlight post‐totality (Pease and Gilbert 2025). Similarly, because habitat structure can shield birds from photic cues, we predicted (P4) that species associated with open habitats (e.g., prairie) would show stronger eclipse responses compared with species that live in dense habitats. Additionally, because migratory species demonstrate activity‐timing flexibility by actively migrating at night, we predicted (P5) that migratory species would show weaker eclipse effects relative to sedentary taxa (Coppack and Bairlein 2011; Zúñiga et al. 2016).

Regarding context, because light pollution motivates diurnal birds to vocalize earlier in the morning and later in the evening than they would otherwise (Gaston et al. 2017; Pease and Gilbert 2025), we predicted (P6) that weaker eclipse effects would emerge in light‐polluted landscapes compared with dark landscapes. Because the highest baseline rates of vocalization typically occur around dawn (Staicer et al. 1996), we predicted (P7) that locations experiencing eclipses earlier in the day would experience stronger suppressive effects than locations experiencing eclipses later in the day when few birds would typically be vocalizing. Finally, because noticeable light obstruction only occurs very near totality, we predicted (P8) that eclipse effects would only emerge at locations with near‐total solar obscuration.

## 2 Methods

### 2.1 Data Collection

Avian vocalization data came from two participatory science efforts, the Eclipse Soundscapes Project and BirdWeather, which both involve automated collection and processing of acoustic data. Sound recordings from both efforts were processed using BirdNET, an artificial neural network trained to identify approximately 6000 species by their vocalizations (Kahl et al. 2021). For the total eclipse, data covered 173 species from 808 locations (269 locations within the path of totality), while the annular eclipse dataset consisted of 85 species from 200 locations (21 locations within the path of annularity).

We collated six species trait variables and three context variables that we hypothesized would mediate bird responses to the eclipse. For traits, we derived two measures of the photic niche (P1): a binary niche classification (nocturnal versus diurnal) from EltonTraits (Wilman et al. 2014) and a continuous niche measure (proportion of nocturnal vocalization) derived from BirdWeather and Eclipse Soundscapes data on non‐eclipse days. We quantified eye size (P2) using the minimum corneal diameter from Stanley Ritland's recently digitized dissertation (Ausprey 2021). We derived a binary nest type (cavity versus not cavity; P3) variable from Birds of the World (Billerman et al. 2020). Habitat density (P4) and migratory classification (P5)—both categorical variables—came from AVONET (Tobias et al. 2022). The context variables were light pollution levels (P6) derived from satellite products at each sensor location (Elvidge et al. 2017), the time of day (relative to sunrise; P7) that a location experienced the eclipse maximum, and the maximum solar obscuration (P8; 0%–100%) that a location experienced during an eclipse, derived using the Eclipse Phase Timing Tool (Winter IIII and Goncalves 2025). For more details, please refer to the Supporting Information.

### 2.2 Comparing Vocalization During Eclipse Maximum to a Normal Day

**FIGURE 2:**
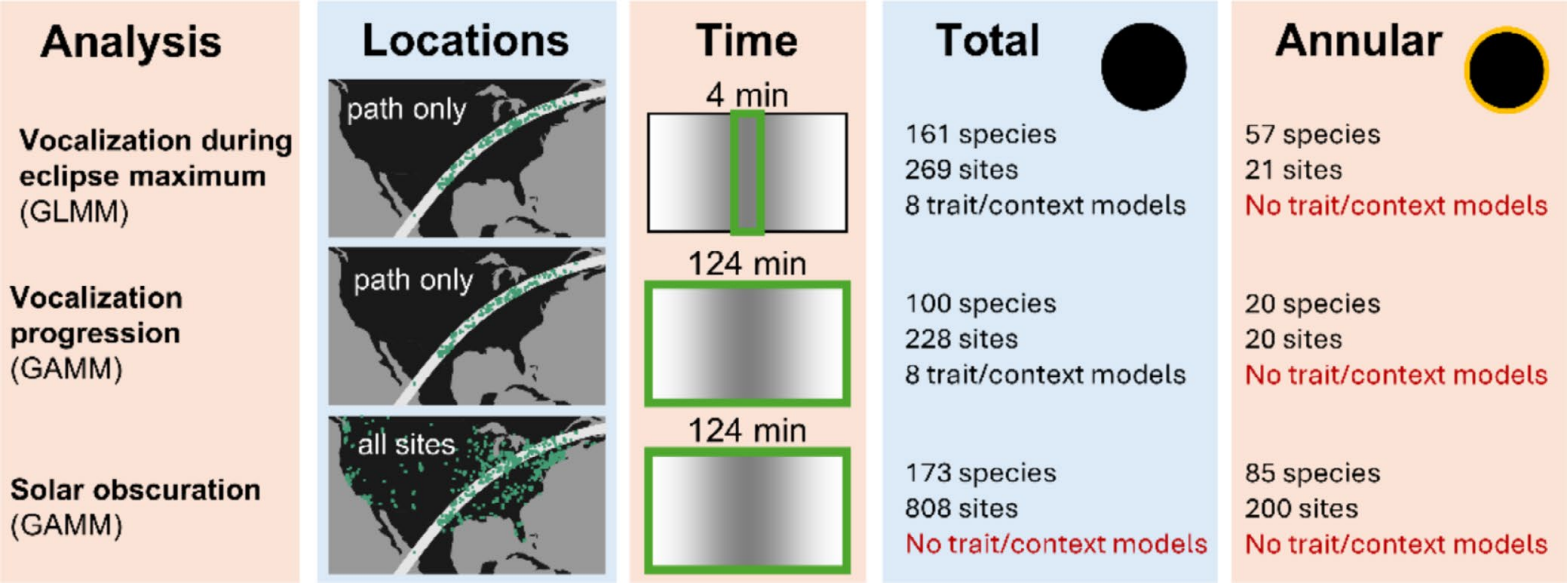
Summary of the three analyses. In the “Time” column, the darkest shading in the center represents eclipse maximum and the green rectangle represents the time window used in each analysis.

**FIGURE 3:**
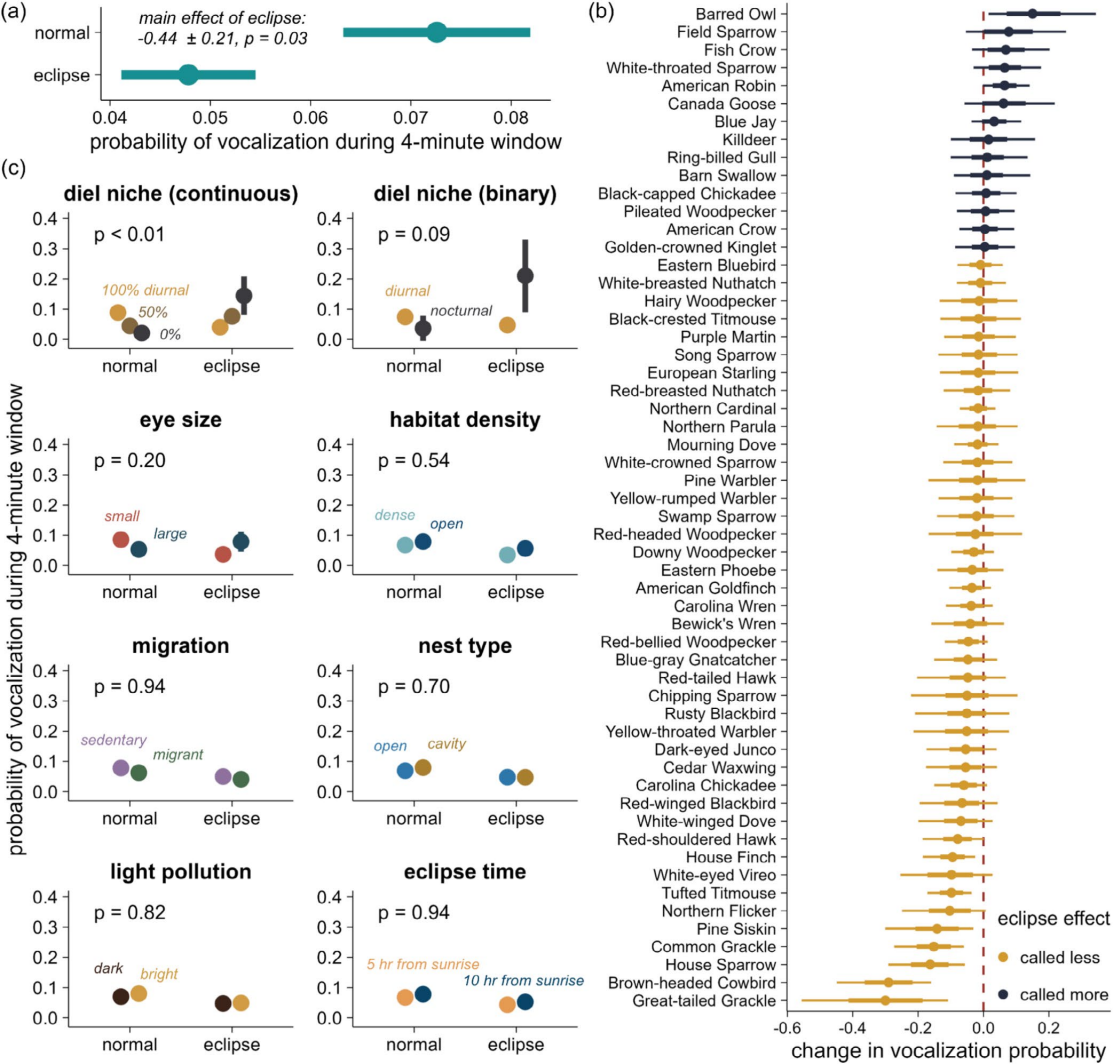
Most species vocalized less during the maximum of the total eclipse. (a) Across species, vocalization probability was lower during totality compared with the same time period on the previous day. Points and bars are point estimates ± one standard error of model predictions. (b) Species‐level differences in vocalization probability during totality (dark: Vocalized more during totality; gold: Vocalized less during totality) relative to the previous day. Points and thick and thin bars are the means and 68% and 95% quantiles of the differences in model predictions for the eclipse versus normal day. (c) A species' photic niche—especially a continuous measure quantifying the proportion of vocalizations during nighttime hours—best explained among‐species variability in eclipse responses. Other trait and context variables did not conclusively predict eclipse effects. The *P*‐value in each panel is the *P*‐value for the interaction between the binary eclipse (normal vs. eclipse) predictor and the trait or context variable. Points and bars are point estimates ± one standard error of model predictions.

To compare vocalizations during eclipse maxima to normal days, we filtered the dataset to retain only stations that were within the eclipse paths (Figure 2). For each station, we defined focal species as those detected within 30 min of the time of eclipse maximum on the day of the eclipse or the preceding day. The response variable was whether (1) or not (0) each species was detected during a 4 min time bin centered on the eclipse maximum for the eclipse day and the same time period on the preceding day. A 4 min window approximates the length of totality for the total eclipse, which varied depending on the position of a location within the path (median: 3.6 min; standard deviation: 0.9 min). Furthermore, we calculated a binary predictor variable indicating whether each observation corresponded to the preceding day (0) or the day of the eclipse (1). This resulted in data for 161 species from 269 unique locations for the total eclipse and 57 species from 21 unique locations for the annular eclipse. We fit generalized linear mixed‐effects models (Bernoulli response with a logit link) to determine the effect of the eclipse on the probability of vocalization. We fit separate models for the annular and total eclipses. For the total eclipse, the model had a random‐effects structure such that the intercept and eclipse predictor varied by species; we also included a random effect for a grouping of species and the 2° grid cell containing the station such that the intercept for each species was adjusted based on location. A Moran's I test indicated there was no evidence (*P* = 0.8) of spatial autocorrelation in model residuals. To evaluate the influence of traits and context, in addition to this base model, we fit a series of 8 models, each of which was the base model with the addition of one trait or context predictor variable, as well as its interaction with the eclipse predictor. For the annular eclipse, we omitted the species–cell random effect due to convergence issues and did not assess the trait models due to the relatively limited number of species sampled during the annular eclipse. We fit models with the glmmTMB package (Brooks et al. 2017) in R and also fit the same models in a Bayesian framework using the brms package (Bürkner 2017) to propagate uncertainty when performing calculations on model predictions to characterize species‐level responses to the eclipse (Figure 3b, Figure S1b).

### 2.3 Characterizing the Progression of Vocalization During Eclipse

The vocalization progression analysis sought to quantify vocalization responses to changing light levels over a broader time window centered on eclipse maximum (Figure 2). We filtered the dataset to retain only stations that were within the eclipse paths. We defined a focal window from 62 min before eclipse maximum to 62 min after eclipse maximum and divided this 124 min window into 31, 4 min bins. We chose 4 min bins because this approximates the length of totality, and we chose the 124 min window so that the central bin would straddle eclipse maximum. Moreover, we reasoned the 124 min window would be broad enough to capture a broad spectrum of ambient light levels as the eclipse progressed (Aguilar et al. 2025) yet narrow enough to avoid dramatic diel‐scale variation in bird vocal behavior (e.g., dawn choruses). We then calculated whether each species was detected (1) or not (0) during each 4 min time bin for a location. We restricted the analysis to species–location combinations for which a species was detected during at least one time bin (i.e., there were no all‐zero detection histories). Moreover, we filtered the dataset to include only species that were detected at least five times total. This resulted in data for 100 species from 228 locations for the total eclipse and 20 species from 20 locations for the annular eclipse. We then fit hierarchical generalized additive models (Pedersen et al. 2019; Wood 2017) with a Bernoulli response and logit link to characterize the progression of vocalization behavior relative to the eclipse (separate models for annular and total). This model—whose commission was to describe cross‐species *and* species‐level vocalization progressions on eclipse day—incorporated a global smoother for time as well as species‐level smoothers with a shared penalty (Pedersen et al. 2019). In addition to this base model, we fit a series of updated models to evaluate the influence of traits and context on vocalization progression. Because the goal of these updated models was to generalize across species‐level idiosyncrasies and quantify how different *types* of species vocalized over time relative to the eclipse, we omitted species‐level time smoothers and instead added a trait or context predictor variable, as well as its interaction with the global smoother. We fit the models using the R package mgcv (Wood 2017).

### 2.4 Quantifying the Influence of Solar Obscuration on Eclipse Responses

Unlike the other analyses, which only used data from within the eclipse paths, we used data from all locations within 3000 km of the centerline of each eclipse path to assess the influence of solar obscuration on vocalization behavior (Figure 2). We took the same approach as the vocalization progression analysis: we calculated whether (1) or not (0) each species was detected during a series of 31, 4 min time bins centered on eclipse maximum. As with vocalization progression analysis, we restricted the analysis to species that were detected at least five times in all. This resulted in data for 173 species from 808 unique locations for the total eclipse and 85 species from 200 unique locations for the annular eclipse. We then fit hierarchical generalized additive models (separate models for annular and total eclipses) with a Bernoulli response and logit link to characterize the progression of vocalization behavior relative to the eclipse and how that progression was influenced by obscuration. The models contained a tensor product smooth between time bin and maximum obscuration that the location experienced (Wood 2017). The models also contained a random intercept for each species–station combination. We fit the models using the R package mgcv (Wood 2017).

## 3 Results

### 3.1 Most Species Vocalized Less During the Total Eclipse; Limited Influence of Annular Eclipse

Across species, birds were less likely to vocalize during the maximum of the total eclipse relative to the same 4 min window on the preceding day (eclipse effect: –0.44 ± 0.21 [standard error], *P* = 0.03; Figure 3a). This equates to a 34.1% decrease (95% credible interval: 52.8%, 8.5%) in vocalization probability during totality relative to the previous day. More species were predicted to show suppressive effects (*n* = 131 species) than promotive effects (*n* = 30 species) of the eclipse. However, there was considerable uncertainty in species‐level responses (Figure 3b). Only seven species showed suppressive eclipse effects with > 95% certainty: Great‐tailed Grackle, Brown‐headed Cowbird, House Sparrow, Common Grackle, Pine Siskin, Tufted Titmouse, and House Finch (Figure 3b; see Table S1 for scientific names). The Barred Owl demonstrated the strongest promotive effect and was the only species to increase in vocalization probability with > 95% certainty (Figure 3b). Notably, this species, while generally considered nocturnal, occasionally vocalizes during the day (Odom and Mennill 2010), particularly on cloudy or rainy days, such that it is colloquially called the “rain owl.” Across species, the annular eclipse did not have a significant association with vocalization probability (eclipse effect: 0.90 ± 2.8 [standard error], *P* = 0.75; Figure S1a). Five species (Clark's Nutcracker, Downy Woodpecker, Cave Swallow, White‐crowned Sparrow, and American Robin) showed increased vocalization during the annular eclipse with > 95% certainty; an additional six species showed increased vocalization with > 68% certainty (Figure S1b).

### 3.2 Photic Niche Traits Predict Total Eclipse Responses but Other Variables Do Not (Total Eclipse Only)

The photic niche of a species strongly predicted whether it vocalized during totality (P1). A continuous measure of a species' photic niche—the proportion of its vocalizations occurring during nighttime hours under normal conditions—strongly predicted eclipse responses: species that vocalize primarily during daylight hours showed a suppressive effect of the eclipse, whereas species that vocalize most intensely before sunrise or after sunset showed promotive effects (eclipse × photic niche interaction: 0.46 ± 0.1, *P* = 0.004; Figure 3c). A binary measure of a species' photic niche (traditional nocturnal vs. diurnal classification) showed similar patterns (nocturnal species showing promotive effects, diurnal species showing suppressive effects) but had a less clear effect (eclipse × photic niche interaction: 2.4 ± 1.6, *P* = 0.09; Figure 3c). Regarding sensory traits, there was a tendency for species with small eyes to show suppressive effects of the eclipse and for species with large eyes to show promotive effects (P2), but the variable was not significant (eclipse × eye size interaction: 0.21 ± 0.17, *P* = 0.20). We documented no statistically significant effects of a species' preferred habitat density (P3; *P* = 0.54), migratory habit (P4; *P* = 0.94), nest type (P5; *P* = 0.70), light pollution at a site (P6; *P* = 0.82), or time (relative to sunrise) of the eclipse (P7; *P* = 0.94; Figure 3c).

### 3.3 Progression of Vocalizations During the Total and Annular Eclipses

**FIGURE 4:**
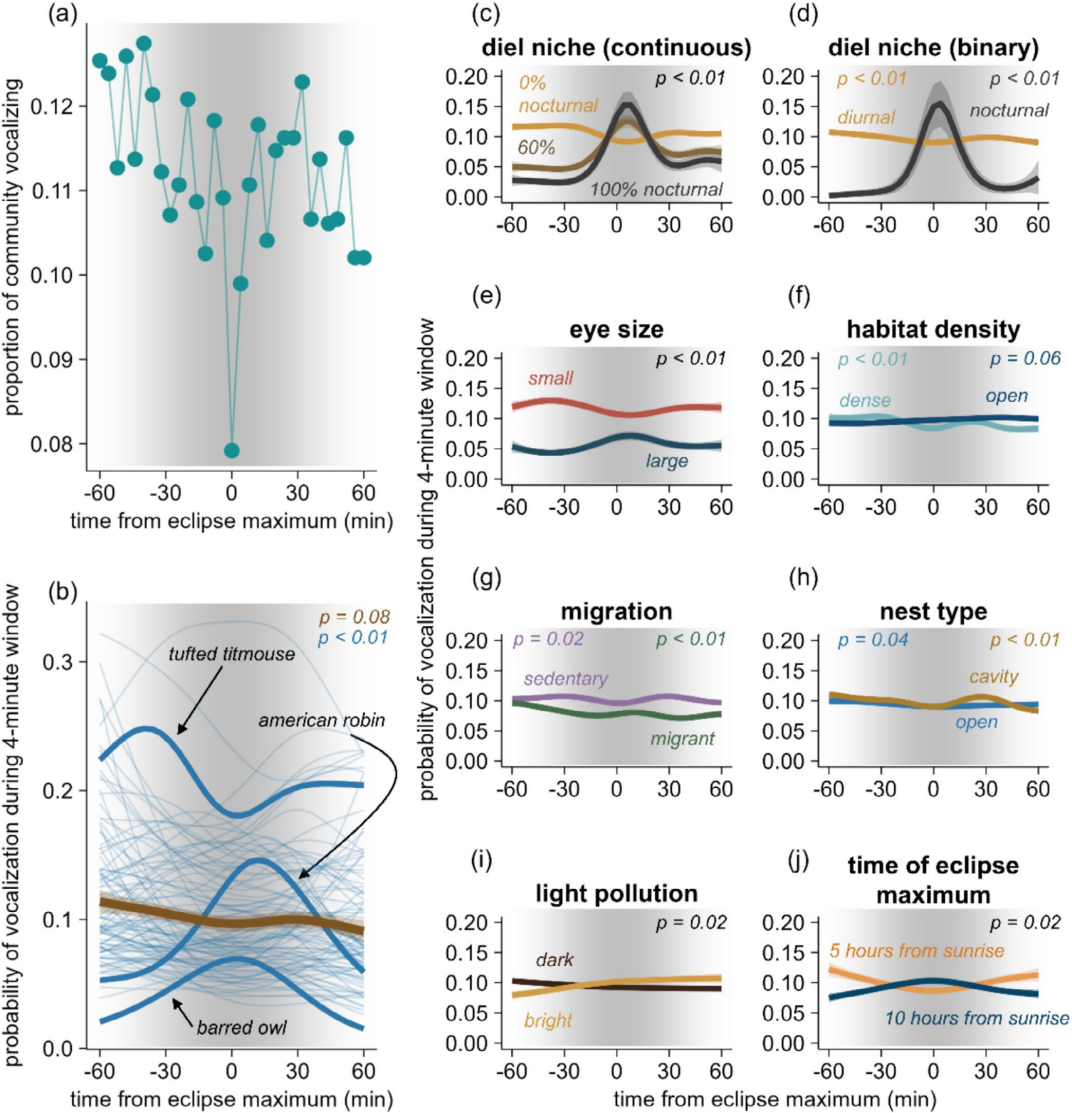
Progression of vocal behavior during the total eclipse. (a) The proportion of species–location combinations with a detection during each 4 min time bin. (b) Model predictions for the progression of vocalization probability averaged across species (brown) and for individual species (blue; bolded lines are species mentioned in the main text). (c–j) Model predictions for the progression of vocalization probability for differing values of the trait and context variables. The gray shading in the background of all panels represents the change in light levels as the eclipse progresses, with darker shades indicating increased solar obscuration. The *P* values represent the significance of a smooth for the interaction between time‐from‐eclipse‐maximum and each trait (separate *P* values for each level of a factor in the case of categorical variables).

The quieting of birds during the total eclipse maximum was ephemeral (Figure 4a). Fewer birds vocalized during totality (8% of species–location combinations) compared with the period immediately before (4 min pre‐totality; 11%) and after (4 min post‐totality, 10%) totality (Figure 4a). However, across species, our model predicted only a negligible decrease in vocalization probability during totality (Figure 4b, brown line). Nonetheless, while the average showed little change, responses varied among species. Eight species showed peaks in vocalization near totality, and the timing of these peaks varied; for example, the Barred Owl was most likely to vocalize 4 min after totality, whereas the American Robin was most likely to vocalize 12 min after totality (Figure 4b). Forty‐four species had the lowest probabilities of vocalization near totality (Figure 4b). For example, the Tufted Titmouse was predicted to show peak vocalization probability 40 min before totality, followed by a “valley” 4 min after totality, and a smaller peak 40 min post‐totality (Figure 4b). Finally, the remaining 48 species did not show peaks or valleys of vocalization near totality; rather, vocalization probability showed general decreases (28 species) or increases (20 species) over the whole 2 h window centered on totality (Figure 4b). In contrast to the total eclipse, the annular eclipse resulted in a slight uptick in vocalization during the 4 min bin centered on maximum (Figure S1c, 17.5% of species–location combinations; the average across all time bins was 11%). Averaged across species, no suppressive or promotive effects emerged at the maximum of the annular eclipse (Figure S1d); however, several species, such as the American Robin, displayed increased vocalization probability during this period (Figure S1d).

### 3.4 Photic Niche and Sensory Traits Predict Vocalization Progression (Total Eclipse Only)

A species' photic niche (P1) better explained among‐species variability in vocalization progression than other trait or context variables, and, as predicted, species associated with dim light conditions were more likely to vocalize near totality than species associated with bright conditions (Figure 4c–j). The continuous measure of a species' photic niche strongly predicted vocalization progression during the total eclipse (*P* < 0.01), outperforming a binary photic niche classification. For example, a species with 60% of its vocalizations occurring at night—like the American Robin—would have a vocalization probability exceeding that of a strictly diurnal species (0% nighttime vocalizations) at 4 min pre‐totality, peaking at 4 min post‐totality, and dropping below that of a strictly diurnal species 20 min post‐totality (Figure 4c). Although a coarser measure, the binary photic niche classification also strongly predicted vocalization progression: diurnal species were predicted to slightly decrease vocalization probability near totality (*P* < 0.01), whereas nocturnal species showed a sharp increase. Eye size predicted vocalization progression (*P* < 0.01): as predicted, small‐eyed species were least likely to vocalize near totality, while large‐eyed species were more likely to vocalize (Figure 4e). Regarding nest type (P3), cavity‐nesters showed a stronger suppressive effect of the eclipse (*P* < 0.01), including the predicted post‐totality increase in vocalization, than species with open nests (*P* = 0.04). Regarding habitat density (P4), species associated with dense habitats vocalized less near totality (*P* < 0.01), while species associated with open habitats showed limited changes in vocalization patterns (*P* = 0.06), opposing our prediction of stronger responses for open‐habitat species. Regarding migration habit (P5), sedentary species tended to show suppressive effects near totality (*P* = 0.02), whereas migratory species showed promotive effects (*P* < 0.01), but the magnitude of these effects did not differ appreciably between groups, opposing our predictions.

Regarding context variables, light pollution levels (P6) did not show the predicted patterns; rather, vocalization probability tended to increase over the entire period in bright landscapes but tended to decrease over the entire period in dark landscapes (*P* = 0.02). Finally, the time of day that a location experienced the eclipse (P7) influenced vocalization progression (*P* = 0.02): in locations where the eclipse occurred early in the day (e.g., 10:00), the predicted suppressive effects of the eclipse were apparent, whereas in locations where the eclipse occurred later in the day (e.g., 15:00), promotive effects emerged such that vocalization probability peaked 4 min pre‐totality (Figure 4j).

### 3.5 Vocalization Peaks at ~80% Obscuration; Suppressive Eclipse Effects Emerge at 94% Obscuration

**FIGURE 5:**
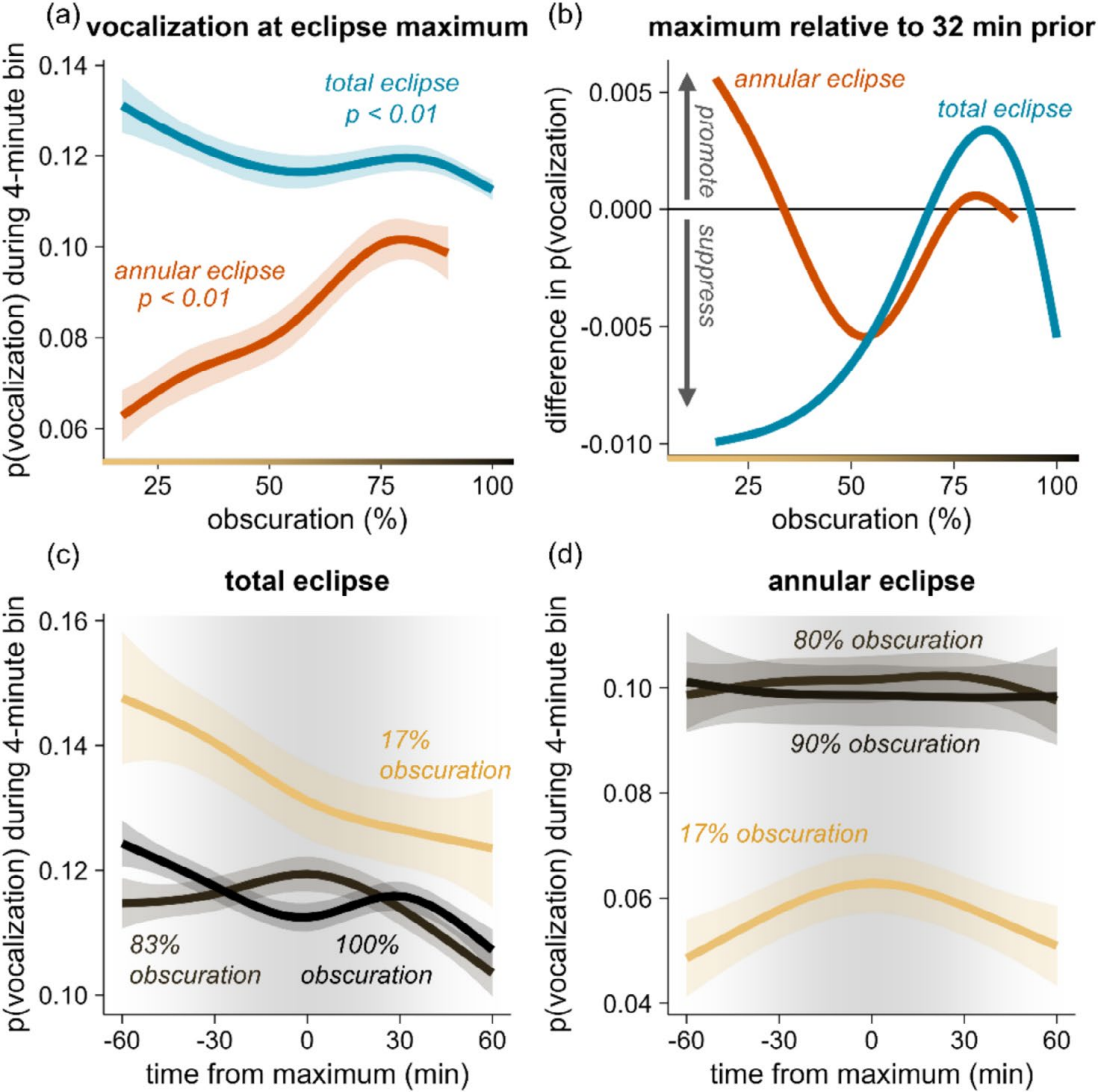
Effects of solar obscuration on avian vocal behavior during the total and annular eclipses. (a) Probability of vocalization during the 4 min time bin centered on eclipse maximum for the across observed values of obscuration. Lines and shaded regions are the means ± one standard error. (b) Difference in vocalization probability at eclipse maximum compared with 32 min pre‐eclipse maximum; positive values represent promotive eclipse effects, whereas negative values represent suppressive eclipse effects. (c–d) Vocalization probabilities for 4 min time bins over a 2 h window centered on eclipse maximum for the total (c) and annular (d) eclipses; colors indicate different levels of obscuration. Lines and shaded regions are means ± one standard error.

Solar obscuration (P8) strongly influenced bird vocalization behavior (*P* < 0.01 for both total and annular eclipses), particularly during the total eclipse (Figure 5). At the total eclipse maximum, vocalization probability declined until approximately 57% obscuration. Vocalization activity then increased to a peak at 81% obscuration before rapidly dropping off (Figure 5a). In contrast, at the annular eclipse maximum, vocalization probability increased until 80% obscuration and subsequently declined as obscuration reached annularity (Figure 5a).

For the total eclipse, at low levels of obscuration (e.g., < 50%), probability of vocalization dropped over the 2 h time window centered on eclipse maximum, likely reflecting the typical quieting of birds through the afternoon (Figure 5c). However, as obscuration increased, birds were more likely to vocalize; at 70% obscuration, promotive effects of the eclipse emerged such that vocalization probability at maximum was the same as vocalization probability 32 min before maximum (Figure 5b). The promotive effect of the total eclipse peaked at 83% obscuration (Figure 5b,c). After 83% obscuration, the promotive effect of the eclipse weakened and switched to a suppressive effect at 94% obscuration (Figure 5b,c). Thus, suppressive effects of the total eclipse on bird vocalizations emerge only at 94% obscuration, which in our dataset occurred at locations within approximately 285 km of the eclipse path centerline.

During the annular eclipse, birds were more likely to vocalize at sites with higher obscuration across the entire two‐hour window centered on maximum (Figure 5d), a result that may emerge because locations with higher obscuration experienced the eclipse closer to sunrise (Pearson's *r* = –0.8). Comparing maximum to 32 min pre‐maximum, promotive effects were apparent between 75% and 87% obscuration (Figure 5b). Because the annular eclipse produced a maximum of only 90% obscuration, the very weak suppressive effects (Figure 5b) are not qualitatively apparent in the plotted model predictions (Figure 5d). Moreover, promotive effects were apparent at lower obscurations (< 33%; Figure 5b,d); this effect may emerge because the locations with low coverage experienced the annular eclipse closer to midday (average eclipse time for locations with < 33% coverage: 6 h after sunrise), when baseline vocalization levels are low.

## 4 Discussion

The sequential dimming and brightening associated with the total eclipse was reflected in avian soundscapes: while the majority of species were less likely to vocalize during the eclipse maximum, this suppressive effect was preceded by *increases* in vocalization, likely the outcome of fading light levels triggering vocal behaviors typically associated with morning and evening twilight. Moreover, considering sites beyond the eclipse paths that varied in the amount of solar obscuration experienced, obscuration levels of ~80% were associated with increases in vocalization; this result appeared for both the total eclipse and the annular eclipse (Figure 5a). This shared pattern between the two eclipses suggests that the dimmer light levels associated with ~80% obscuration may serve as behavioral thresholds in many species. In contrast, only the total eclipse showed suppressive effects, with suppressive effects emerging at ~94% obscuration, indicating that near‐complete darkness is required to cause most species to fall silent (the annular eclipse only reached 90% obscuration). Reinforcing the importance of the photic niche concept, photic niche measures—and to a lesser degree, eye size (Figure 4e)—were the traits that most clearly predicted species responses to eclipses.

A continuous measure of a species' photic niche (the proportion of its vocalizations occurring before sunrise or after sunset under typical conditions) more strongly predicted species' eclipse responses than any other trait or context variable evaluated, including a traditional nocturnal/diurnal classification. Strikingly, many ostensibly diurnal species frequently vocalized at night (La 2012), including the American Robin (60% of all detections before sunrise or after sunset), Killdeer (54%), and Horned Lark (40%). Given the proliferation of round‐the‐clock, automated data collection, together with emerging efforts to measure in situ light levels (BirdWeather 2024) and new statistical methods to quantify species' diel niches (Gerber et al. 2024; Iannarilli et al. 2025), the time is ripe for the addition of photic niche traits to existing avian trait databases (Tobias et al. 2022). Such photic niche traits could inform species' sensory adaptations (Dominoni et al. 2020), foraging niche (Ausprey 2021), interactions with other species (Post 2019), and responses to light pollution (Davies and Smyth 2018; Pease and Gilbert 2025) and weather conditions (Cohen et al. 2020; Levy et al. 2019). Key challenges to adding photic niche traits to the arsenal of existing trait databases include obtaining data for species that occur in locations poorly sampled by participatory science programs (Culumber et al. 2019; Rosário et al. 2025) and quantifying inevitable seasonal variation in diel activity patterns arising from species‐specific phenologies of reproductive behavior and territoriality which produce seasonal variation in the timing of vocalization (Staicer et al. 1996).

While solar eclipses happen approximately every 18 months somewhere on Earth, they are rare at any given location and it is thus difficult to provide generalities about their effects on wildlife behavior. Timing—on both seasonal and diel scales—is perhaps the most important context germane to wildlife behavior. At least in temperate zones, many bird species show seasonal pulses in the vigor of vocalization, reflecting territory defense and mate attraction (Staicer et al. 1996). This pattern emerges when comparing the results for the total eclipse (which occurred during the breeding season for many species included in our analysis) and the annular eclipse (which occurred in the non‐breeding season): baseline levels of vocalization were lower on the day of the annular eclipse than the day of the total eclipse (Figure 5c,d). We therefore speculate that suppressive effects on vocalization are more apparent for eclipses that occur during the breeding season, simply because of the higher rates of baseline vocalization during these seasons. While we did not find evidence for time‐of‐day effects when focusing only on the eclipse maximum (Figure 3c), when stepping back to evaluate the entire 2 h period centered on maximum (Figure 4j), locations experiencing the eclipse earlier in the day showed suppressive effects, whereas locations experiencing the eclipse later in the day showed promotive effects. This likely reflects diel patterns of bird vocalization: earlier in the day, birds are vocalizing more vigorously, and therefore suppressive effects may be more apparent. Additionally, or alternatively, eclipses that occur later in the day (e.g., 10 h after sunrise; Figure 4j) may align more closely with birds' circadian clockwork for commencing evening vocalization, producing a promotive effect. The accumulation and archiving of data from additional eclipses will allow for a better understanding of the temporal context of eclipse effects.

Participatory science programs like the Eclipse Soundscapes Project and BirdWeather leverage the power of the people to provide observations from many locations representing many species (Oestreich et al. 2025). While such broad‐scale acoustic datasets can reveal insights into vocalization patterns, they simply cannot document what birds are doing during eclipses other than vocalizing—or not vocalizing. Under the assumption that vocalization correlates with other types of activity, it is tempting to conclude that the silence of diurnal species indicates inactivity, perhaps roosting, whereas the vocalizations of nocturnal species indicate the initiation of foraging and other activities. Testing these assumptions will require exploring other datastreams. Radar data—another broad‐scale datastream—can quantify the volume of animals flying, and since many species migrate at night (Van Doren and Horton 2018) or fly to communal roosts at sunset (Perez et al. 2024), additional analyses of radar data (Nilsson et al. 2018) may add nuance to the story we document, with the caveat that species (or indeed broader taxonomic groupings like birds versus bats) cannot be differentiated from these data. A key opportunity lies in the analysis of individual‐based data such as GPS trackers (Kays et al. 2015), accelerometers (Brown et al. 2013), and RFID systems deployed at locations like nests or feeders that anchor behaviors of interest (Madsen et al. 2021). In some cases, existing data‐collection programs such as Motus (Taylor et al. 2017) may provide coincidental data for eclipses that have already happened; alternatively, researchers may develop and deploy targeted behavioral observation systems to provide fine‐grained measurements of individual birds during future eclipses. All told, while it is easy to conceive limitations of acoustic participatory science programs, it is worth reflecting on how far we have come in understanding animal responses to eclipses in less than 100 years: from hand‐written notes of observations made by human eyes and years, delivered by mail, to continuously recording, standardized listening devices and processing algorithms delivering by cloud and email. As disparate as the two approaches are, it is perhaps remarkable that a common story emerges: many bird species, except those associated with dim light levels, are less likely to vocalize during totality.

## Author Contributions

Neil A. Gilbert: conceptualization (equal), formal analysis (equal), investigation (equal), methodology (equal), project administration (equal), software (equal), supervision (equal), visualization (equal), writing – original draft (equal), writing – review and editing (equal). Brent S. Pease: conceptualization (equal), data curation (equal), formal analysis (equal), investigation (equal), methodology (equal), project administration (equal), resources (equal), software (equal), supervision (equal), validation (equal), visualization (equal), writing – original draft (equal), writing – review and editing (equal). MaryKay Severino: conceptualization (equal), funding acquisition (equal), project administration (equal), supervision (equal), writing – review and editing (equal). Henry ‘Trae’ Winter III: conceptualization (equal), data curation (equal), funding acquisition (equal), project administration (equal), supervision (equal), writing – review and editing (equal).

## Funding

This work was supported by the National Aeronautics and Space Administration, 80NSSC21M0008.

## Conflicts of Interest

The authors declare no conflicts of interest.

## Supporting information


**Data S1:** ece373090‐sup‐0001‐Supinfo.docx.

## Data Availability

Data and code to reproduce the study are archived on Zenodo: https://doi.org/10.5281/zenodo.15790879.
