# Supplementary material for: Photic Niche Explains Avian Behavioral Responses to Solar Eclipses
Source: Ecol Evol. 2026 Feb 10;16(2):e73090. doi: 10.1002/ece3.73090 (PMC12891433; doi:10.1002/ece3.73090)
Supplement: Supplementary file 1 — Data S1: ece373090‐sup‐0001‐Supinfo.docx. [file ECE3-16-e73090-s001.docx]

**Photic niche explains avian behavioral responses to solar eclipses**

**Supporting Information**

Our sampling focused on the annular eclipse of 14 October 2023 (Fig. 1b of the main text) and the total eclipse on 8 April 2024 (Fig. 1c of the main text), both of which were experienced across a large geographic swathe of North America. During an annular eclipse, the moon blocks most, but not all, of the Sun’s light at annularity (approximately 90% maximum solar obscuration in this case), contrasting with a total solar eclipse, which reaches 100% solar obscuration at totality, when the moon completely blocks the surface of the sun from view. We refer to the time at which solar obscuration reaches its highest value at a given location as **eclipse maximum**. Only sites within the path of annularity in 2023 or the path of totality in 2024 experienced these peak levels of obscuration (Fig. 1b–c of the main text). All other sites experienced a partial eclipse, where eclipse maximum did not reach the levels of annularity or totality. During the October 14, 2023 annular eclipse, the path of annularity, the narrow area where people could witness the “ring of fire,” stretched from Oregon across the western and southwestern United States to Texas, continuing into Central and South America, and was was approximately 125 miles (210 km) wide (Fig. 1b). During the April 8, 2024 total solar eclipse, the path of totality, the narrow region where people could experience complete sun coverage, stretched from Mexico across the central and northeast United States, from Texas to Maine, before continuing into Canada, and was approximately 108 to 122 miles (175 to 200 km) wide (Fig. 1c).

Volunteers collected data through two participatory science efforts: the Eclipse Soundscapes Project and BirdWeather. The Eclipse Soundscapes Project is a NASA Citizen Science project funded by NASA Science Activation that uses participatory sciences to study how eclipses affect life on Earth. Eclipse Soundscapes volunteers deployed AudioMoth acoustic loggers (version 1.2.0) developed by Open Acoustic Devices [(Hill *et al.* 2019)](https://www.zotero.org/google-docs/?bFOKUE) to collect sound recordings. AudioMoths were programmed to record 24-hours/day for a total of 5 days: two days before, during, and two days after the eclipse. AudioMoths were equipped with 3 AA batteries and an onboard 64GB MicroSD card for storing audio recordings. The sampling rate was set to 48 kHz and a medium gain was used. The AudioMoths were housed in heavy-duty plastic bags. AudioMoths were deployed by volunteers at a sampling location of their choice.

We additionally used BirdWeather, a living library of bird vocalizations [(BirdWeather 2024)](https://www.zotero.org/google-docs/?07SCse) to assess bird vocalization behavior during the eclipses. BirdWeather is an open-access, volunteer-contributed program that collects acoustic recordings primarily with Raspberry Pis—inexpensive single-board computers [(Raspberry Pi 2020)](https://www.zotero.org/google-docs/?O3PJw7)—or Portable Universal Codecs (PUCs). While Raspberry Pis were designed for indoor use, PUCs are weatherproof acoustic recording units with onboard environmental sensors and an edge neural engine. BirdWeather sensors operated 24-hours/day continuously without regard to the eclipse, so using BirdWeather’s data explorer we downloaded North America data during October 2023 and April 2024 and subset the data to the sample sampling periods as the Eclipse Soundscapes Project.

Sound recordings from the Eclipse Soundscapes Project and BirdWeather were processed using BirdNET, an artificial neural network trained to identify approximately 6,000 species by their vocalizations [(Kahl *et al.* 2021)](https://www.zotero.org/google-docs/?W6vvhZ). BirdNET converts audio recordings into 3- (default) or 6-second spectrograms and predicts the top three most likely species using a species list generated by the metadata of the recording (time of year and location). The resulting BirdNET output includes a species identification and an associated confidence score that ranges from 0 to 1, which is a probability measure of whether the vocalization was labeled correctly, with greater values indicating an increased probability of a reliable label.

Like all convolutional neural networks, BirdNET classifications may include false positives and false negatives (mean overall precision = 0.78). Ideally, species-specific confidence thresholds would be used to minimize classification errors, but a comprehensive set of such thresholds is not currently available. Given the broad taxonomic and geographical scope of our analysis, we applied a uniform confidence threshold of 0.65 across all species. Uniform confidence thresholds between 0.5 and 0.75 are commonly applied in studies using BirdNET [(Kahl *et al.* 2021; Wood *et al.* 2021)](https://www.zotero.org/google-docs/?5SZQUn), ideally balancing the trade-off between reducing false positives and retaining true positive detections. We used the default sensitivity, overlap, and bandpass filter settings of BirdNET (1.0, 0, 0–15,000 respectively).

We also collated 6 species trait variables and 2 context variables that we hypothesized would influence eclipse responses. The 6 traits were diel niche (2 measures), eye size, nest type, habitat association, and migratory classification, while the 2 context variables were light pollution levels at the site and the time of day that a site experienced the eclipse. First, we obtained a binary measure of a species’ diel niche (nocturnal versus diurnal) from EltonTraits [(Wilman *et al.* 2014)](https://www.zotero.org/google-docs/?TEprBt). Second, we calculated a continuous measure of a species’ diel niche using acoustic data from BirdWeather and Eclipse Soundscapes; we calculated this variable for April 2024 only, since we did not conduct trait/context analyses for the annular eclipse due to the limited number of species and locations sampled. When calculating the diel niche, we omitted data from date of the eclipses and thus had 4 days of Eclipse Soundscapes data (two days prior to the eclipse, two days after the eclipse) and 14 days of BirdWeather data (we downloaded 15 days of BirdWeather data centered on the eclipse dates). We retrieved sunrise and sunset times for each date and location represented in the data using the R package suncalc [(Thieurmel & Elmarhraoui 2022)](https://www.zotero.org/google-docs/?HWn2gr). We then calculated the proportion of detections for each species that occurred during the nighttime (i.e., before sunrise or after sunset). Third, we obtained eye size measurements from Stanley Ritland’s unpublished dissertation from the 1980’s that were recently digitized and published [(Ausprey 2021)](https://www.zotero.org/google-docs/?RbrIQ6). The database has measurements for 4,782 individual birds representing 3,497 species. The dataset provides multiple measures of eye size, which are highly correlated, and thus we chose the “CD1” measurement (minimum corneal diameter). Eye size measurements were available for 127/161 (79%) of species in our dataset; for the remaining 34 (21%) of our species that were not represented in the Ritland dataset, we imputed the mean value for the family (all families were represented in the Ritland dataset). Eye size shows strong patterns of phylogenetic conservation [(Ausprey 2021)](https://www.zotero.org/google-docs/?NajRF7), and thus we believe this is a reasonable solution to the incomplete species-level coverage of the Ritland database. Next, we obtained species-level habitat density scores (dense, semi-open, open) and migratory classification (sedentary, partial migrant, migrant) from the AvoNET database [(Tobias *et al.* 2022)](https://www.zotero.org/google-docs/?9sjfIw). We additionally created a binary nest type variable indicating whether a species nests in cavities (1) or not (0). We did so by reviewing the “Breeding” page of each species’ Birds of the World account and coded a species as a cavity-nester if it typically nests in tree cavities, burrows, or other similar opaque chamber [(Billerman *et al.* 2020)](https://www.zotero.org/google-docs/?ifkDyd). In addition to these species traits, we calculated two context variables: light pollution levels at each sensor location and the time of day (relative to sunrise) that a location experienced the eclipse. To calculate light pollution, we extracted radiance corresponding to sensor coordinates and the month of observations from the Visible Infrared Imaging Radiometer Suite (VIIRS) Day/Night Band [(Elvidge *et al.* 2017)](https://www.zotero.org/google-docs/?xD0xd8). This data product quantifies global radiance associated with human activities (moonlight and natural sources of light such as fires and volcanoes are omitted) at 15 arc second spatial resolution (~500m at the Equator) and monthly temporal resolution. Finally, we calculated eclipse time by subtracting local sunrise time—retrieved using the R package suncalc [(Thieurmel & Elmarhraoui 2022)](https://www.zotero.org/google-docs/?AqM26U)—from the peak eclipse time for each location.

**References**

[Ausprey, I.J. (2021). Adaptations to light contribute to the ecological niches and evolution of the terrestrial avifauna. *Proceedings of the Royal Society B: Biological Sciences*, 288, 20210853.](https://www.zotero.org/google-docs/?RKWqcq)

[Billerman, S., Keeney, B., Rodewald, P. & Schulenberg, T. (2020). Birds of the World. *Cornell Laboratory of Ornithology, Ithaca, NY, USA*.](https://www.zotero.org/google-docs/?RKWqcq)

[BirdWeather. (2024). *BirdWeather: a living library of bird vocalizations*. Available at: https://www.birdweather.com/. Last accessed .](https://www.zotero.org/google-docs/?RKWqcq)

[Elvidge, C.D., Baugh, K., Zhizhin, M., Hsu, F.C. & Ghosh, T. (2017). VIIRS night-time lights. *International journal of remote sensing*, 38, 5860–5879.](https://www.zotero.org/google-docs/?RKWqcq)

[Hill, A.P., Prince, P., Snaddon, J.L., Doncaster, C.P. & Rogers, A. (2019). AudioMoth: A low-cost acoustic device for monitoring biodiversity and the environment. *HardwareX*, 6, e00073.](https://www.zotero.org/google-docs/?RKWqcq)

[Kahl, S., Wood, C.M., Eibl, M. & Klinck, H. (2021). BirdNET: A deep learning solution for avian diversity monitoring. *Ecological Informatics*, 61, 101236.](https://www.zotero.org/google-docs/?RKWqcq)

[Raspberry Pi. (2020). Raspberry Pi Annual Review 2020.](https://www.zotero.org/google-docs/?RKWqcq)

[Thieurmel, B. & Elmarhraoui, A. (2022). *suncalc: Compute Sun Position, Sunlight Phases, Moon Position and Lunar Phase*.](https://www.zotero.org/google-docs/?RKWqcq)

[Tobias, J.A., Sheard, C., Pigot, A.L., Devenish, A.J.M., Yang, J., Sayol, F., *et al.* (2022). AVONET: morphological, ecological and geographical data for all birds. *Ecology Letters*, 25, 581–597.](https://www.zotero.org/google-docs/?RKWqcq)

[Wilman, H., Belmaker, J., Simpson, J., de la Rosa, C., Rivadeneira, M.M. & Jetz, W. (2014). EltonTraits 1.0: Species-level foraging attributes of the world’s birds and mammals: Ecological Archives E095-178. *Ecology*, 95, 2027–2027.](https://www.zotero.org/google-docs/?RKWqcq)

[Wood, C.M., Kahl, S., Chaon, P., Peery, M.Z. & Klinck, H. (2021). Survey coverage, recording duration and community composition affect observed species richness in passive acoustic surveys. *Methods in Ecology and Evolution*, 12, 885–896.](https://www.zotero.org/google-docs/?RKWqcq)

**
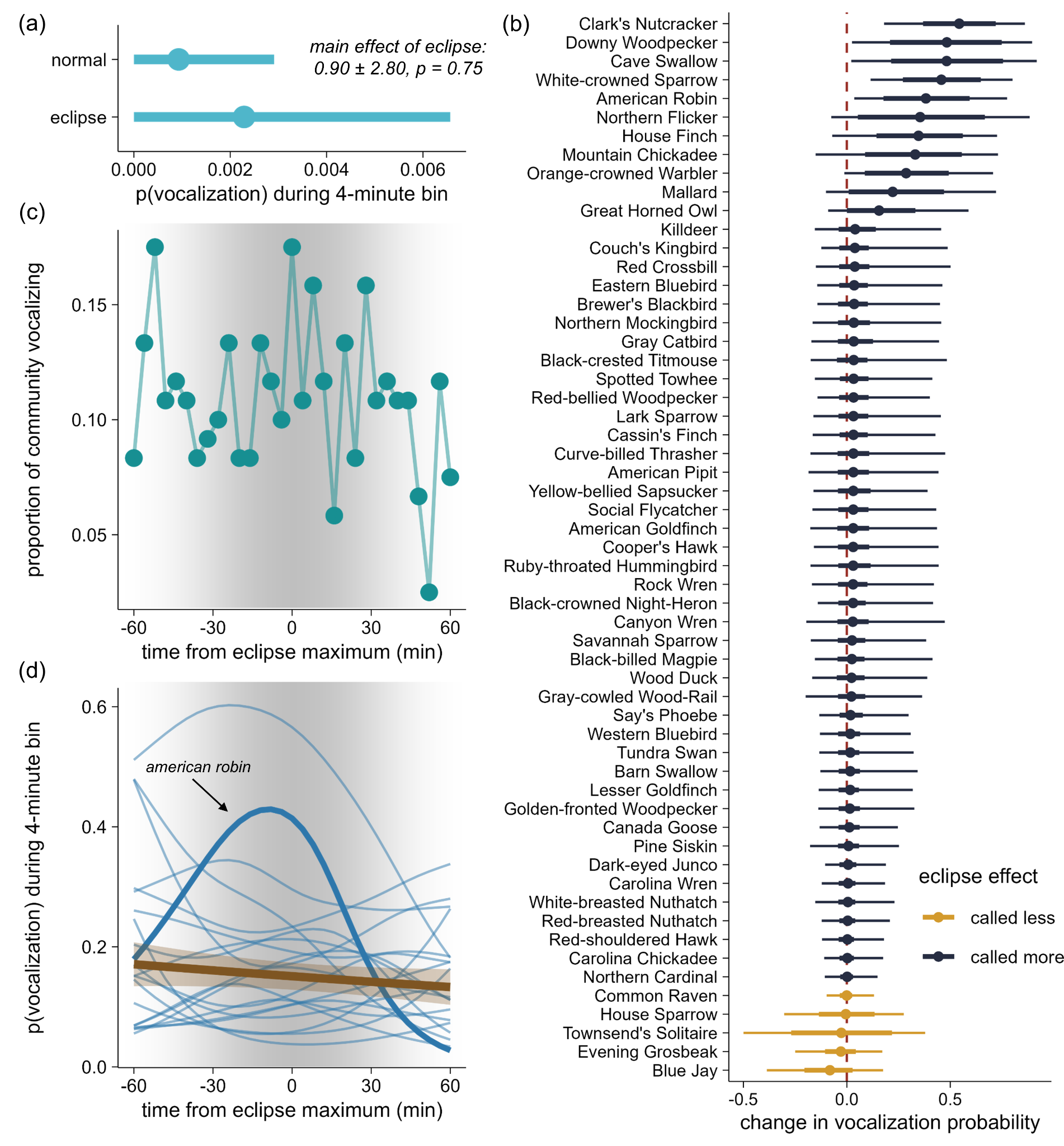
**

**Figure S1. Behavioral responses of birds to the 14 October 2023 annular eclipse.** (a) Across species, there was no evidence of changes in vocalization behavior during the maximum of the annular eclipse compared to the same time on the preceding day. Points and bars are point estimates ± one standard error of model predictions. (b) Species-level differences in vocalization probability during the maximum of the annular eclipse relative to the previous day (dark: called more during eclipse; gold: called less during eclipse). Points and thick and thin bars are the means and 68% and 95% quantiles of the differences in model predictions for the eclipse versus normal day. (c) The proportion of species–location combinations with a detection for each 4-minute time bin. (d) Progression of vocalization probability across species (brown) and for individual species (blue; bolded lines are species mentioned in the main text).

Table S1. Species included in analyses. The ‘eclipse’ column reports whether data for the species was included for both eclipses, the total eclipse only, or the annular eclipse only.

| common | scientific | eclipse |
| --- | --- | --- |
| Inca Dove | *Columbina inca* | total |
| White-winged Dove | *Zenaida asiatica* | total |
| Eurasian Collared-Dove | *Streptopelia decaocto* | both |
| Gila Woodpecker | *Melanerpes uropygialis* | both |
| House Sparrow | *Passer domesticus* | both |
| Northern Rough-winged Swallow | *Stelgidopteryx serripennis* | total |
| Green-winged Teal | *Anas crecca* | total |
| Orange-crowned Warbler | *Leiothlypis celata* | both |
| Fish Crow | *Corvus ossifragus* | total |
| Northern Mockingbird | *Mimus polyglottos* | both |
| Eastern Phoebe | *Sayornis phoebe* | both |
| Greater Yellowlegs | *Tringa melanoleuca* | total |
| Mourning Dove | *Zenaida macroura* | both |
| Northern Cardinal | *Cardinalis cardinalis* | both |
| Red-bellied Woodpecker | *Melanerpes carolinus* | both |
| Belted Kingfisher | *Megaceryle alcyon* | both |
| Boat-tailed Grackle | *Quiscalus major* | total |
| Carolina Wren | *Thryothorus ludovicianus* | both |
| Common Grackle | *Quiscalus quiscula* | both |
| Eastern Bluebird | *Sialia sialis* | both |
| Pileated Woodpecker | *Dryocopus pileatus* | both |
| Great-tailed Grackle | *Quiscalus mexicanus* | both |
| House Finch | *Haemorhous mexicanus* | both |
| Osprey | *Pandion haliaetus* | total |
| Rock Pigeon | *Columba livia* | total |
| Western Kingbird | *Tyrannus verticalis* | total |
| Blue-gray Gnatcatcher | *Polioptila caerulea* | total |
| Northern Parula | *Setophaga americana* | total |
| Yellow-throated Vireo | *Vireo flavifrons* | total |
| American Goldfinch | *Spinus tristis* | both |
| Killdeer | *Charadrius vociferus* | both |
| Laughing Gull | *Leucophaeus atricilla* | total |
| Purple Martin | *Progne subis* | total |
| Chimney Swift | *Chaetura pelagica* | total |
| Cactus Wren | *Campylorhynchus brunneicapillus* | total |
| Couch's Kingbird | *Tyrannus couchii* | total |
| Curve-billed Thrasher | *Toxostoma curvirostre* | both |
| Pyrrhuloxia | *Cardinalis sinuatus* | total |
| Red-shouldered Hawk | *Buteo lineatus* | both |
| Red-winged Blackbird | *Agelaius phoeniceus* | total |
| Ash-throated Flycatcher | *Myiarchus cinerascens* | total |
| Hooded Oriole | *Icterus cucullatus* | total |
| Merlin | *Falco columbarius* | total |
| Say's Phoebe | *Sayornis saya* | both |
| Bell's Vireo | *Vireo bellii* | total |
| American Crow | *Corvus brachyrhynchos* | both |
| Eastern Meadowlark | *Sturnella magna* | total |
| Great Crested Flycatcher | *Myiarchus crinitus* | total |
| Black-bellied Whistling-Duck | *Dendrocygna autumnalis* | total |
| Yellow-headed Blackbird | *Xanthocephalus xanthocephalus* | total |
| Barn Swallow | *Hirundo rustica* | both |
| Brown-headed Cowbird | *Molothrus ater* | total |
| Black-throated Sparrow | *Amphispiza bilineata* | total |
| Bewick's Wren | *Thryomanes bewickii* | both |
| Scissor-tailed Flycatcher | *Tyrannus forficatus* | total |
| Summer Tanager | *Piranga rubra* | total |
| Verdin | *Auriparus flaviceps* | total |
| Cedar Waxwing | *Bombycilla cedrorum* | both |
| European Starling | *Sturnus vulgaris* | both |
| Carolina Chickadee | *Poecile carolinensis* | both |
| Downy Woodpecker | *Dryobates pubescens* | both |
| American Kestrel | *Falco sparverius* | both |
| Blue Jay | *Cyanocitta cristata* | both |
| Blue-headed Vireo | *Vireo solitarius* | total |
| Ruby-crowned Kinglet | *Corthylio calendula* | total |
| Tufted Titmouse | *Baeolophus bicolor* | both |
| Black-crested Titmouse | *Baeolophus atricristatus* | both |
| Ladder-backed Woodpecker | *Dryobates scalaris* | total |
| White-eyed Vireo | *Vireo griseus* | total |
| Lesser Goldfinch | *Spinus psaltria* | both |
| Cave Swallow | *Petrochelidon fulva* | both |
| Barred Owl | *Strix varia* | total |
| Broad-winged Hawk | *Buteo platypterus* | total |
| Ruby-throated Hummingbird | *Archilochus colubris* | total |
| White-crowned Sparrow | *Zonotrichia leucophrys* | both |
| Black-and-white Warbler | *Mniotilta varia* | total |
| Pine Siskin | *Spinus pinus* | both |
| Eastern Kingbird | *Tyrannus tyrannus* | total |
| Song Sparrow | *Melospiza melodia* | both |
| Pine Warbler | *Setophaga pinus* | total |
| Field Sparrow | *Spizella pusilla* | total |
| Canyon Wren | *Catherpes mexicanus* | total |
| Common Raven | *Corvus corax* | both |
| Yellow-rumped Warbler | *Setophaga coronata* | both |
| American Robin | *Turdus migratorius* | both |
| Brown Thrasher | *Toxostoma rufum* | total |
| White-throated Sparrow | *Zonotrichia albicollis* | both |
| Ring-billed Gull | *Larus delawarensis* | both |
| Wood Duck | *Aix sponsa* | total |
| Chipping Sparrow | *Spizella passerina* | total |
| Cooper's Hawk | *Accipiter cooperii* | both |
| Eastern Towhee | *Pipilo erythrophthalmus* | total |
| Prothonotary Warbler | *Protonotaria citrea* | total |
| Red-headed Woodpecker | *Melanerpes erythrocephalus* | total |
| Yellow-throated Warbler | *Setophaga dominica* | total |
| Prairie Warbler | *Setophaga discolor* | total |
| Anna's Hummingbird | *Calypte anna* | both |
| Brewer's Sparrow | *Spizella breweri* | total |
| Gambel's Quail | *Callipepla gambelii* | both |
| Northern Flicker | *Colaptes auratus* | both |
| Black-chinned Hummingbird | *Archilochus alexandri* | total |
| Red-breasted Nuthatch | *Sitta canadensis* | both |
| Red-tailed Hawk | *Buteo jamaicensis* | both |
| Caspian Tern | *Hydroprogne caspia* | total |
| Louisiana Waterthrush | *Parkesia motacilla* | total |
| Black Phoebe | *Sayornis nigricans* | both |
| Black-throated Gray Warbler | *Setophaga nigrescens* | total |
| Bushtit | *Psaltriparus minimus* | both |
| California Towhee | *Melozone crissalis* | both |
| Nuttall's Woodpecker | *Dryobates nuttallii* | both |
| Abert's Towhee | *Melozone aberti* | total |
| Marsh Wren | *Cistothorus palustris* | total |
| Wilson's Warbler | *Cardellina pusilla* | total |
| American Woodcock | *Scolopax minor* | total |
| Allen's Hummingbird | *Selasphorus sasin* | total |
| Lawrence's Goldfinch | *Spinus lawrencei* | total |
| Pacific-slope Flycatcher | *Empidonax difficilis* | total |
| Western Bluebird | *Sialia mexicana* | both |
| Common Yellowthroat | *Geothlypis trichas* | total |
| Mallard | *Anas platyrhynchos* | both |
| Savannah Sparrow | *Passerculus sandwichensis* | total |
| Dark-eyed Junco | *Junco hyemalis* | both |
| Hutton's Vireo | *Vireo huttoni* | total |
| Spotted Towhee | *Pipilo maculatus* | both |
| Wrentit | *Chamaea fasciata* | total |
| California Scrub-Jay | *Aphelocoma californica* | both |
| Oak Titmouse | *Baeolophus inornatus* | both |
| Great Horned Owl | *Bubo virginianus* | both |
| Hooded Warbler | *Setophaga citrina* | total |
| Brown-headed Nuthatch | *Sitta pusilla* | both |
| White-breasted Nuthatch | *Sitta carolinensis* | both |
| Costa's Hummingbird | *Calypte costae* | total |
| Brown Creeper | *Certhia americana* | both |
| Winter Wren | *Troglodytes hiemalis* | total |
| Golden-crowned Kinglet | *Regulus satrapa* | both |
| Mountain Chickadee | *Poecile gambeli* | both |
| Purple Finch | *Haemorhous purpureus* | total |
| Black-throated Green Warbler | *Setophaga virens* | total |
| Black-capped Chickadee | *Poecile atricapillus* | both |
| Pygmy Nuthatch | *Sitta pygmaea* | both |
| Steller's Jay | *Cyanocitta stelleri* | both |
| Canada Goose | *Branta canadensis* | both |
| Wild Turkey | *Meleagris gallopavo* | total |
| Bald Eagle | *Haliaeetus leucocephalus* | both |
| Tree Swallow | *Tachycineta bicolor* | both |
| Swamp Sparrow | *Melospiza georgiana* | total |
| Chestnut-backed Chickadee | *Poecile rufescens* | both |
| Acorn Woodpecker | *Melanerpes formicivorus* | both |
| Hairy Woodpecker | *Dryobates villosus* | both |
| Golden-crowned Sparrow | *Zonotrichia atricapilla* | both |
| Violet-green Swallow | *Tachycineta thalassina* | total |
| Yellow-bellied Sapsucker | *Sphyrapicus varius* | both |
| Rusty Blackbird | *Euphagus carolinus* | total |
| Townsend's Warbler | *Setophaga townsendi* | total |
| Common Redpoll | *Acanthis flammea* | total |
| Brewer's Blackbird | *Euphagus cyanocephalus* | total |
| American Coot | *Fulica americana* | total |
| Gadwall | *Mareca strepera* | total |
| Black-billed Magpie | *Pica hudsonia* | both |
| American Tree Sparrow | *Spizelloides arborea* | total |
| Common Goldeneye | *Bucephala clangula* | total |
| Eurasian Tree Sparrow | *Passer montanus* | total |
| Tundra Swan | *Cygnus columbianus* | total |
| Sandhill Crane | *Antigone canadensis* | total |
| Horned Lark | *Eremophila alpestris* | total |
| Trumpeter Swan | *Cygnus buccinator* | total |
| Fox Sparrow | *Passerella iliaca* | total |
| Herring Gull | *Larus argentatus* | total |
| American Wigeon | *Mareca americana* | total |
| Ring-necked Pheasant | *Phasianus colchicus* | total |
| Varied Thrush | *Ixoreus naevius* | total |
| Western Meadowlark | *Sturnella neglecta* | total |
| Pacific Wren | *Troglodytes pacificus* | both |
| MacGillivray's Warbler | *Geothlypis tolmiei* | annular |
| California Quail | *Callipepla californica* | annular |
| Clark's Nutcracker | *Nucifraga columbiana* | annular |
| Evening Grosbeak | *Coccothraustes vespertinus* | annular |
| Townsend's Solitaire | *Myadestes townsendi* | annular |
| Hermit Thrush | *Catharus guttatus* | annular |
| Greater White-fronted Goose | *Anser albifrons* | annular |
| Cackling Goose | *Branta hutchinsii* | annular |
